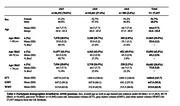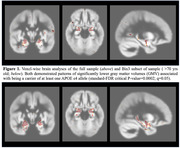# Gray Matter Alterations Associated with Aging & APOEε4 Genotype in 37,437 Participants from the UK Biobank

**DOI:** 10.1002/alz.089560

**Published:** 2025-01-09

**Authors:** Emma J Gleave, Paul M. Thompson, Priya Rajagopalan

**Affiliations:** ^1^ Imaging Genetics Center, Mark and Mary Stevens Neuroimaging & Informatics Institute, University of Southern California, Marina del Rey, CA USA; ^2^ Imaging Genetics Center, Mark and Mary Stevens Neuroimaging and Informatics Institute, Keck School of Medicine, University of Southern California, Marina del Rey, CA USA; ^3^ Imaging Genetics Center, Mark and Mary Stevens Neuroimaging and Informatics Institute, University of Southern California, Marina Del Rey, CA USA

## Abstract

**Background:**

Carrying one or more copies of the apolipoprotein E ε4 allele represents the greatest known source of genetic risk for late‐onset Alzheimer’s disease (AD), although the mechanisms are not fully understood. Several smaller‐scale studies have analyzed APOEε4 effects on brain volume, reporting gray matter volume (GMV) alterations in medial temporal and precuneal regions in people with AD and in healthy APOEε4 carriers. Here, we analyzed brain images from a large sample of healthy elderly adults in relation to ε4 carrier status, stratifying by age to assess potential group differences.

**Method:**

The Computational Anatomy Toolbox (CAT12) was used to perform a large‐scale voxelwise segmentation of 3D T1‐weighted brain MRI data from the UK Biobank. The statistical relationship between voxel‐wise brain GMV and APOEε4 genotype was assessed using a dominant model for presence or absence of APOEε4 allele across 37,437 subjects (51.7% female; 64.6 (7.7SD) years old), separated into three age bins (Bin1: <60 yrs old; Bin2: 60‐70 yrs old; Bin3: >70 yrs old; Table 1). Linear mixed model analyses for APOEε4 in relation to GMV were performed for the full sample and within each age bin, adjusting for age, sex, genetic ancestry, intracranial volume (ICV), and scan site.

**Result:**

Lower GMV was associated with APOEε4 (standard‐FDR critical P‐value=0.0002; q=0.05), after covarying for age, sex, ancestry, and ICV. Effects were detected in the bilateral mesial temporal lobe structures including bilateral amygdala, posterior hippocampus and punctate foci within bilateral thalami. A large region of association was found in the oldest age‐group (>70yrs; Bin3). Significance maps are shown in Figure 1.

**Conclusion:**

We identified alterations in GMV related to carrying at least one APOEε4 allele in one of the largest neuroimaging samples to date. Extending prior work, even healthy adult carriers of the APOEε4 haplotype demonstrated neurostructural gray matter alterations. Such neurostructural alterations may be more salient in older age, highlighting the need for continued study of ε4 expression mechanisms and their interaction with aging